# The Dual Role of Zinc Homeostasis in Gram-Negative Bacterial Pathogenicity and Host Immune Defense

**DOI:** 10.3390/microorganisms14081633

**Published:** 2026-07-27

**Authors:** Yueting Cai, Yuankai Yan, Ning Shen, Jingyuan Liu, Chunjing Du

**Affiliations:** 1 Peking University Health Science Center, Peking University, Beijing 100191, China; 2210122616@stu.pku.edu.cn (Y.C.); 2210301354@stu.pku.edu.cn (Y.Y.); 2 Department of Critical Care Medicine, Peking University Ditan Teaching Hospital, Beijing 100015, China; 3 Center of Infectious Disease, Peking University Third Hospital, Beijing 100191, China; shenningputh@163.com; 4 Department of Pulmonary and Critical Care Medicine, Peking University Third Hospital, Beijing 100191, China; 5 Department of Critical Care Medicine, Capital Medical University Affiliated Beijing Ditan Hospital, Beijing 100015, China

**Keywords:** zinc, Gram-negative bacteria, zinc homeostasis, nutritional immunity, bacterial virulence, host–pathogen interactions

## Abstract

Gram-negative bacteria pose a significant threat to global health due to their high prevalence, multidrug resistance, and association with various infections. Zinc, an essential trace element, plays a dual role in host–microbe interactions, being crucial for both host immune function and bacterial metabolism. Maintaining optimal zinc homeostasis is essential for effective host defense and microbial survival. We comprehensively examine the multifaceted roles of zinc in host–microbe interactions, particularly focusing on its implications for the pathogenicity of Gram-negative bacteria and host immunity. As part of nutritional immunity, hosts have evolved zinc-mediated antimicrobial strategies that manipulate metal availability at the host–pathogen interface, including zinc limitation that restricts bacterial access to this essential nutrient and zinc intoxication that exposes invading bacteria to cytotoxic zinc concentrations. In Gram-negative bacteria, zinc homeostasis, maintained through zinc transporters and efflux systems, critically governs bacterial virulence, biofilm formation, and survival under host-imposed nutritional immunity. By integrating current advances, this review highlights zinc homeostasis as a central determinant of host–pathogen interactions and a promising target for combating multidrug-resistant Gram-negative infections. Understanding these complex zinc-mediated mechanisms provides new perspectives for developing metal-targeted therapeutic strategies.

## 1. Introduction

Gram-negative bacteria represent a significant and persistent threat to global public health due to their high prevalence and association with a wide spectrum of infections, including pneumonia, urinary tract infections, bloodstream infections, and gastrointestinal diseases [1,2]. The urgency of addressing these pathogens is underscored by their remarkable capacity to develop multidrug resistance, exemplified by the increasing incidence of infections caused by critical-priority pathogens such as *Klebsiella pneumoniae*, *Pseudomonas aeruginosa*, and *Acinetobacter baumannii*, as classified by World Health Organization (WHO). The high morbidity and mortality rates associated with these infections, particularly in healthcare settings and among immunocompromised individuals, highlight the severe clinical and economic burdens they impose.

The pathogenicity of Gram-negative bacteria is mediated by an array of virulence factors that facilitate host colonization, immune evasion, and tissue damage [3]. These factors include lipopolysaccharide (LPS), which triggers potent inflammatory responses; secretion systems (e.g., Type III secretion systems) that inject effector proteins into host cells; adhesins for attachment; and capsules that protect against phagocytosis [4,5,6]. In response, the host employs sophisticated innate and adaptive immune mechanisms to detect and eliminate invading pathogens. An emerging host response in this interaction is “nutritional immunity”—a host antimicrobial strategy that manipulates the availability of essential trace metals at the host–pathogen interface, including both metal restriction and metal intoxication, thereby impairing microbial growth and survival [7,8]. Conversely, pathogens have evolved intricate mechanisms to overcome host-imposed metal deprivation and resist metal intoxication.

Among these essential nutrients, zinc plays a dual and pivotal role in host–microbe conflicts. It is a key micronutrient involved in both host immune function and bacterial metabolism, where it serves as a cofactor for numerous enzymes and transcriptional regulators [7,9,10]. This review provides a comprehensive overview of zinc’s biological functions, regulatory mechanisms and its interactions with the host and Gram-negative bacteria. Understanding the multifaceted roles of zinc in both bacterial pathogenicity and host immunity is crucial for developing novel therapeutic strategies to combat the growing threat of multidrug-resistant Gram-negative bacterial infections.

## 2. The Biological Functions and Significance of Zinc in the Host Immunity and Gram-Negative Bacteria

Zinc is an essential trace element in living organisms, involved in a wide range of fundamental physiological processes, including growth, immune regulation, and biomolecular synthesis such as proteins and nucleic acids. Understanding the regulatory mechanisms of zinc homeostasis in the host and bacteria is beneficial for maintaining host health and combating bacterial infections.

### 2.1. The Biological Functions of Zinc

The biological functions of zinc are described as follows. Firstly, zinc functions as a catalytic and structural cofactor for numerous metalloenzymes and regulatory proteins involved in DNA replication, protein synthesis, and cell-cycle control, thereby contributing to genomic stability and mitotic fidelity. In addition, it participates in multiple cellular processes, including signal transduction, proliferation, differentiation, and apoptosis, and helps maintain intracellular homeostasis [11,12]. Secondly, zinc participates in immune regulation by modulating the proliferation, differentiation, and activation of immune cells, including macrophages, T cells, and B cells, and by supporting antibody production and immunological memory formation [13,14]. Thirdly, zinc contributes to the regulation of gene expression by serving as a structural cofactor for zinc finger proteins (ZFPs), stabilizing their characteristic tertiary structures and enabling these sequence-specific transcription factors to recognize and bind cognate DNA elements [15]. Fourthly, beyond its role in enzymes involved in DNA and protein synthesis, zinc also acts as a catalytic or structural cofactor by binding to the active sites of more than 300 enzymes, including metabolic enzymes, digestive enzymes, and oxidoreductases, thereby contributing to enzymatic activity, cellular metabolism, energy production, and redox homeostasis [16]. Finally, zinc helps preserve cellular redox homeostasis by stabilizing biological membranes and ion channels, protecting protein sulfhydryl groups from oxidation, and regulating antioxidant pathways such as metallothionein expression and Cu/Zn-superoxide dismutase (SOD) activity [17,18].

### 2.2. The Effects of Zinc Deficiency/Excess on the Host Immunity

**Figure 1:**
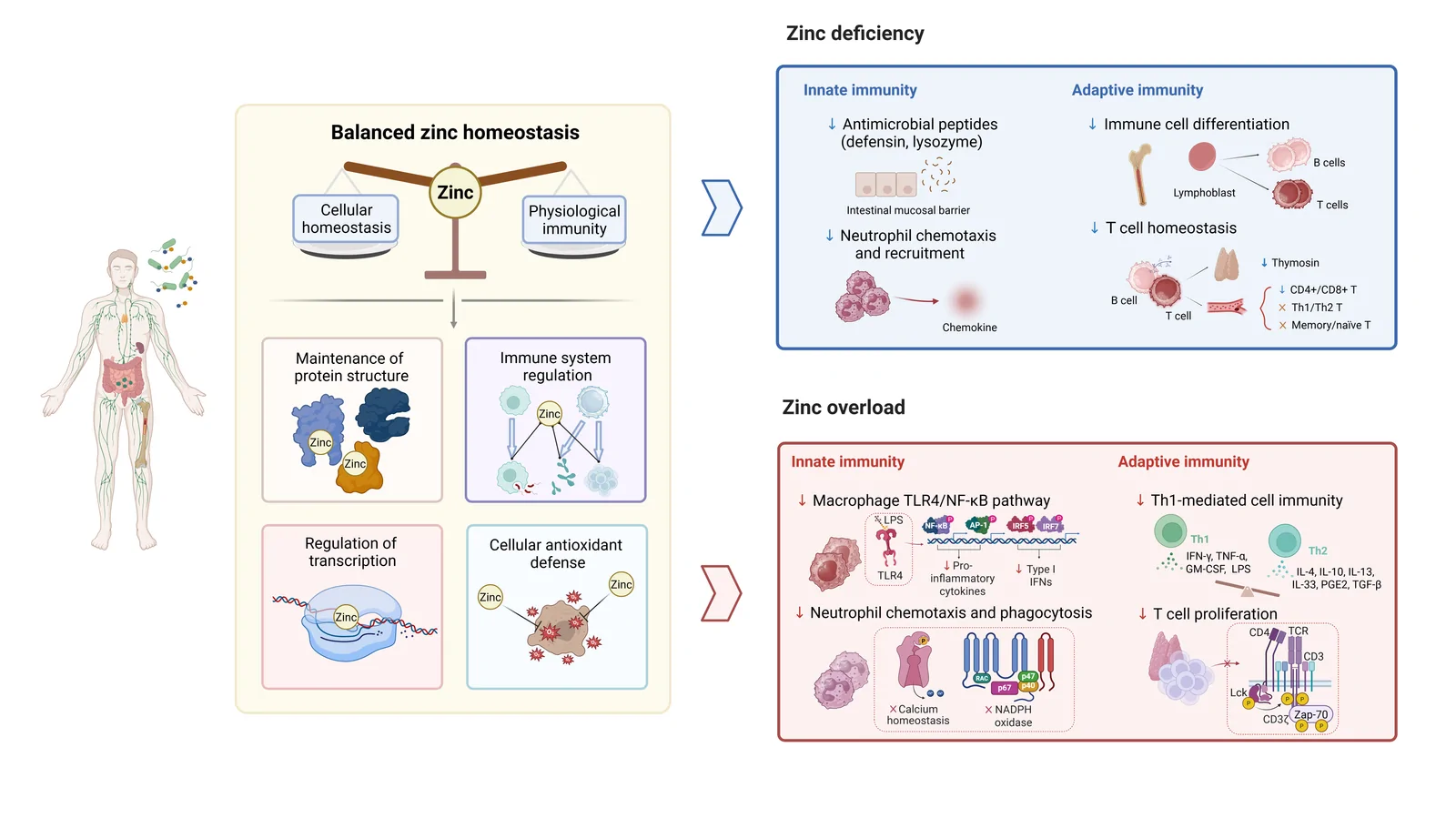
Biological functions of zinc and its bidirectional effects on host immune system. Zinc is essential for maintaining protein structure, regulating gene transcription, modulating immune responses, and supporting antioxidant defense. Both zinc deficiency and overload disrupt immune homeostasis. Its deficiency impairs innate immunity by reducing antimicrobial peptide production and diminishing neutrophil chemotaxis. It also compromises adaptive immunity by impairing thymosin synthesis and disrupting the balance among CD4^+^/CD8^+^, Th1/Th2, and naïve/memory T cells. Zinc overload, conversely, suppresses immune activity by inhibiting macrophage TLR4/NF-κB signaling and reducing pro-inflammatory cytokine release. It also weakens phagocytosis and chemotaxis of neutrophils by disrupting calcium homeostasis and NADPH oxidase. Excess zinc also impairs adaptive immunity by inhibiting Th1-mediated cellular response and reducing T-cell proliferation. For example, its overload inhibits Lck kinase activity and suppresses CD3 zeta chain (CD3ζ) phosphorylation, thereby blocking TCR signaling. Together, maintaining balanced zinc homeostasis is essential for optimal immune defense and prevention of both immunodeficiency and hyperinflammatory disorders.

Zinc is an essential trace element involved in a wide range of physiological processes, including immune regulation, growth, wound repair, and enzyme-mediated biochemical reactions. Both deficiency and excess of zinc can have significant effects on the host (Figure 1).

Zinc deficiency can disrupt multiple physiological processes, particularly those related to immune function. In innate immunity, chronic zinc deficiency can impair the integrity of the intestinal mucosal barrier by reducing antimicrobial peptide synthesis of intestinal epithelial cells (e.g., defensins, lysozyme) [19,20]. It also affects the expression of chemokines and their related receptors. This impairs chemotaxis and recruitment of innate immune cells such as neutrophils, limiting the body’s ability to clear pathogens and increasing infection risk [21,22]. For adaptive immunity, zinc deficiency impairs the development and function of both T and B cells, with more severe effects on T cells. Through shared pathways, its deficiency disrupts the normal development and maturation of T and B lymphocyte progenitor cells in the bone marrow. In T cells, the thymus provides the microenvironment for T lymphocyte differentiation and maturation, with thymosin involved in this process. Zinc deficiency is associated with impaired thymic function and reduced thymosin production, which can compromise T cell development and T cell-mediated immunity. It also disrupts T cell homeostasis, including the balance of naïve and memory T cells, the CD4^+^/CD8^+^ ratio, and the balance between Th1 and Th2 helper T cells [23,24]. For example, chronic diarrhea leading to zinc loss, if not replenished in time, can severely disrupt T cell homeostasis, impairing defense against infections and immune memory [25].

Excessive zinc intake can disturb trace-element homeostasis, particularly copper metabolism, and is associated with impaired immune function. Daily intake exceeding 50 mg of zinc for more than 12 weeks can induce copper deficiency in humans, resulting in anemia and neurological impairment [26]. Disruption of trace-element homeostasis can also affect immune function. At the innate immune level, supraphysiological zinc concentrations have been reported to suppress the phagocytic and microbicidal activities of leukocytes, thereby reducing antimicrobial defense [27]. At concentrations above 100 μM, zinc ions attenuate macrophage inflammatory responses by inhibiting the TLR4/NF-κB signaling pathway and reducing the production of pro-inflammatory cytokines, including TNF-α and IL-1β [28]. Exposure to high zinc reduces neutrophil chemotaxis and phagocytic efficiency by approximately 30~50%, while simultaneously disrupting intracellular Ca^2+^ flux and NADPH oxidase activity [29]. Natural killer cells and dendritic cells likewise exhibit functional suppression when extracellular zinc reaches 100 μM [30]. Paradoxically, despite zinc’s well-documented anti-inflammatory and antioxidant properties, its chronic excessive exposure is associated with persistent low-grade inflammation, as supraphysiological zinc levels can promote inflammatory responses through multiple mechanisms [31,32]. At still higher concentrations, zinc can aberrantly activate NF-κB while simultaneously inhibiting histone deacetylases (HDACs), driving monocytes to sustain the release of pro-inflammatory mediators such as IL-6 and TNF-α [29]. This collapse of anti-inflammatory homeostasis can precipitate a cytokine storm and markedly amplify the inflammatory cascade. Zinc overload also disturbs adaptive immunity. In a recent animal study, mice fed a high-zinc diet exhibited a pronounced shift in the Th1/Th2 balance, with IFN-γ falling by 40% and IL-4 rising 1.8-fold [29,33]. This result showed the redirection of immune response from Th1-mediated cellular immunity toward Th2-mediated humoral immunity. Excess zinc can suppress T lymphocyte activation and proliferation by interfering with TCR signaling. At high intracellular concentrations, zinc has been reported to inhibit lymphocyte-specific protein tyrosine kinase (LCK) activity and reduce phosphorylation of the CD3 zeta chain (CD3ζ), thereby impairing proximal TCR signal transduction. In addition, intracellular zinc selectively suppresses downstream ERK1/2 phosphorylation after CD3-mediated TCR stimulation [34,35]. Above all, maintaining optimal zinc intake is essential to systemic immunity.

### 2.3. The Effects of Zinc Deficiency/Excess on Gram-Negative Bacteria

Zinc is a biologically important micronutrient in bacteria, where it participates in diverse processes, including enzyme function, protein structural stabilization, gene regulation, and stress responses. Perturbations in its availability, whether limitation or excess, can affect bacterial physiology, metabolism, and adaptation.

Zinc limitation can impair bacterial fitness by affecting the structural stability and functional activity of zinc-dependent proteins and enzymes. At the molecular level, zinc deficiency compromises bacterial envelope integrity and structural maintenance, leading to morphological defects and heightened sensitivity to oxidative and environmental stresses [36]. Zinc serves as a cofactor for many bacterial enzymes involved in metabolism and growth. Gram-negative bacteria have evolved high-affinity zinc uptake systems to acquire zinc under metal-limiting conditions. ZnuABC is a broadly conserved high-affinity zinc uptake system in the vast majority of Gram-negative bacteria and is typically upregulated under zinc-limited conditions [37,38,39,40]. Deletion of this system reduces bacterial fitness and affect virulence-related traits, including biofilm formation and, in motile Gram-negative species, flagellar gene expression and motility [38,41,42,43,44]. At the transcriptional level, zinc limitation induces zinc-scavenging and zinc-sparing programs, including high-affinity zinc uptake systems and the expression of zinc-independent ribosomal paralogues RpmE2 and RpmJ2, which replace the zinc-binding ribosomal proteins RpmE (bL31) and RpmJ (bL36), respectively [45,46]. Changes in zinc availability can also affect iron-homeostasis and oxidative-stress regulons, as shown by Fur- and SoxS-related transcriptional responses during the transition from zinc starvation to zinc repletion in *E. coli* [47,48]. Its deficiency weakens antioxidant defense systems and reduces bacterial resilience to stressors like antibiotics.

Although zinc is essential for bacterial physiology, it imposes metal stress and exerts antibacterial effects by perturbing metal homeostasis, interfering with enzyme activity and compromising stress tolerance when in excess. Zinc toxicity is mainly mediated by mismetallation, competition with other transition metals, and interference with metalloenzymes rather than direct redox cycling [49,50]. However, bacteria can mount adaptive metal-resistance responses to counteract zinc overload and maintain metal homeostasis. For example, in *P. aeruginosa*, excess zinc induces the P-type ATPase CadA and the CzcCBA efflux system, while deletion of *cadA* or *czcA* increases susceptibility to zinc stress [51]. In *E. coli*, zinc overload perturbs iron and copper homeostasis, increases cellular iron demand, decreases copper tolerance, and inhibits Fe-S cluster biogenesis by targeting Fe–S assembly proteins, thereby impairing Fe–S-dependent enzyme activity and contributing to growth inhibition under zinc stress [52,53]. Similarly, in *S. enterica* serovar Typhimurium, loss of the zinc efflux systems ZntA and ZitB increases susceptibility to zinc overload and nitric oxide-mediated nitrosative stress, causing growth delay upon nitric oxide exposure, toxic intracellular zinc accumulation in activated macrophages, and a competitive disadvantage in nitric oxide-producing mice [54]. Excess zinc can also weaken bacterial stress tolerance and compromise antibiotic resistance. The zinc ionophore PBT2 has demonstrated antibiotic-resensitizing activity across multiple Gram-negative pathogens [55]. In *K. pneumoniae*, PBT2-mediated zinc accumulation suppresses SOD activity, increases reactive oxygen species (ROS) production, and disrupts cell-wall synthesis, thereby reversing tigecycline resistance [56].

Beyond direct metal toxicity, zinc-based interventions also influence antibiotic-associated phenotypes by modulating bacterial stress responses, mutagenesis, horizontal gene transfer, and biofilm-associated tolerance. The bacterial SOS response is a DNA damage-induced regulatory program that can promote error-prone repair and accelerate the emergence of antibiotic resistance under antibiotic stress [57]. Zinc salts have been reported to suppress SOS-induced hypermutation in *E*. *coli* and *K*. *pneumoniae*, while zinc pyrithione more potently inhibits ciprofloxacin-induced hypermutation in *Enterobacter cloacae* [58,59,60]. Zinc also reduced extended-spectrum β-lactamase gene transfer in Enterobacteriaceae [59]. Moreover, zinc oxide nanoparticles combined with meropenem substantially reduced the meropenem MIC and biofilm biomass of carbapenem-resistant *K*. *pneumoniae*, with downregulation of biofilm-associated genes such as *mrkA*, *fimA*, and *ecpA* [61]. These findings support the potential use of zinc-based materials as adjunctive antimicrobial strategies. Overall, as in the host, bacterial intracellular zinc levels must be maintained within a narrow physiological range. Its limitation or excess can disrupt metal homeostasis, thereby impairing bacterial growth, metabolism and stress adaptation.

## 3. Regulation of Zinc Homeostasis in the Host and Gram-Negative Bacteria

**Figure 2:**
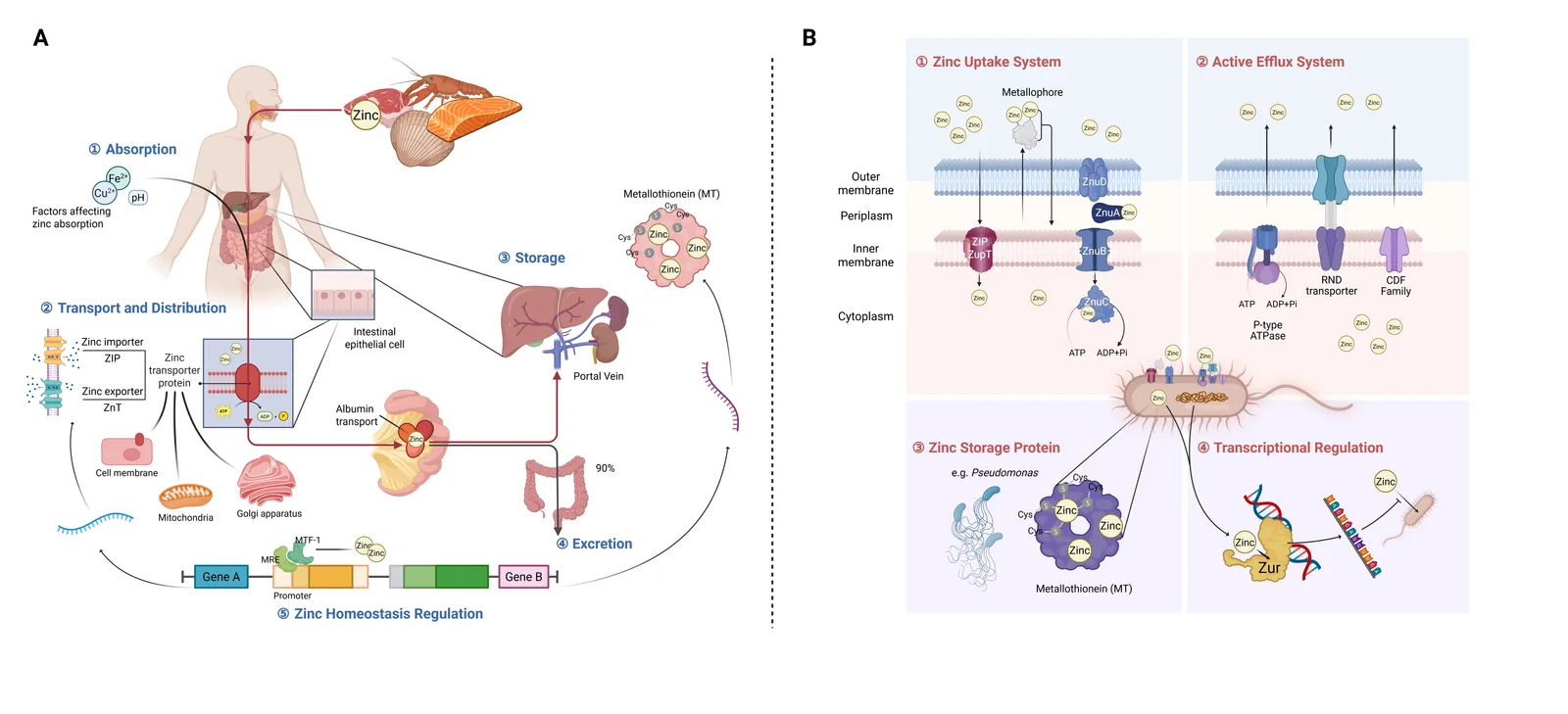
Regulation of zinc homeostasis in the host and Gram-negative bacteria. Zinc homeostasis is tightly regulated in both the host and Gram-negative bacteria to sustain physiological balance. (**A**) In the host, dietary zinc is absorbed mainly in the small intestine, influenced by factors such as competing ions (Fe^2+^, Cu^2+^, Ca^2+^) and luminal pH. Following intestinal absorption, zinc is transported through the portal circulation in association with albumin and other circulating ligands, while its transmembrane movement is regulated by ZIP importers and ZnT exporters. Intracellular zinc is buffered by metallothioneins (MTs) and stored in organelles such as mitochondria and the Golgi apparatus. Excess zinc is excreted primarily via the gastrointestinal tract. Zinc homeostasis is transcriptionally regulated by metal-regulatory transcription factor 1 (MTF-1), which activates genes containing metal response element (MRE) to maintain zinc equilibrium. (**B**) In Gram-negative bacteria, zinc uptake is mediated by the low-affinity ZupT system and the high-affinity ZnuABC system under deficiency, while excess zinc is expelled via P-type ATPases, RND transporters, and CDF proteins. The high-affinity zinc uptake system involves metallophore-associated acquisition, recognition by outer-membrane receptors such as ZnuD, periplasmic zinc binding by ZnuA, inner-membrane translocation through the ZnuB permease, and ATP hydrolysis driven by the ZnuC ATPase. Among efflux systems, P-type ATPases and CDF-family proteins primarily mediate zinc export across the inner membrane, whereas RND transporters form envelope-spanning efflux systems across the inner membrane, periplasm, and outer membrane. The genus *Pseudomonas* provides a representative example of bacterial metallothionein-mediated zinc storage, in which intracellular MTs sequester excess zinc. The zinc uptake regulator (Zur) senses intracellular bioavailable zinc through reversible Zn^2+^ binding to its regulatory metal-binding sites. Under zinc-replete conditions, Zn^2+^-bound Zur binds Zur-box sequences and represses zinc acquisition genes, whereas zinc limitation reduces Zur metallation, weakens Zur–DNA binding, and derepresses zinc uptake and zinc-sparing responses to restore zinc homeostasis.

The regulation of zinc homeostasis is a critical process in both the host and Gram-negative bacteria, playing a key role in immune responses, bacterial survival, and infection dynamics (Figure 2).

### 3.1. Regulation of Zinc Homeostasis in the Host

In the host, zinc levels are controlled through dietary intake, intestinal absorption, systemic distribution, and excretion. Zinc is absorbed primarily in the small intestine, and its absorption efficiency is influenced by dietary composition, competing ions such as Fe^2+^, Ca^2+^, and Cu^2+^, intestinal pH, and its chemical form [62,63]. Once absorbed, it is distributed throughout the body. Zinc is predominantly intracellular, with approximately 85% located in muscles and bones, 11% in the skin and liver, and the remaining 4% distributed among other tissues including ocular tissues such as the retina and choroid, as well as the prostate and kidneys [28,64,65]. Within cells, it is found in the nucleus (30~40%), cytosol, organelles, and associated with cell membranes [66,67]. Zinc transporters, belonging to the SLC30 (ZnT) and SLC39 (ZIP) families, transport zinc across biological membranes and help maintain intracellular and intra-organellar zinc homeostasis [68] (Table 1). ZnT transporters generally export zinc from the cytoplasm or into intracellular compartments, while ZIP transporters import zinc into the cytoplasm from the extracellular space or intracellular stores [69]. Additional transport pathways involve calcium channels and neurotransmitter receptors [70,71]. Intracellular zinc storage in the host is predominantly mediated by metallothioneins (MTs). These small, cysteine-rich proteins can bind multiple zinc ions, buffering intracellular free zinc levels and safeguarding against potential toxicity while ensuring zinc availability when needed [72]. MTs are involved in zinc binding, movement within the cell, and transportation to various cellular compartments. Zinc can also be stored in cellular vesicles, sometimes referred to as “zincosomes”, and in secretory vesicles for exocytosis [73]. The endoplasmic reticulum and Golgi apparatus are also significant intracellular zinc stores [74]. Cellular zinc levels are monitored by metal-regulatory transcription factor 1 (MTF-1), which senses zinc and activates genes involved in zinc regulation, such as those encoding MTs and zinc transporters, ensuring zinc remains within physiological thresholds [75,76]. Excess zinc is excreted primarily through the kidneys and gastrointestinal tract. The kidneys play a key role in regulating plasma zinc levels by filtering and reabsorbing zinc as needed [77]. The liver also contributes to zinc homeostasis by MTs to store and release zinc in response to systemic demands [78].

### 3.2. General Regulatory Framework of Zinc Homeostasis in Gram-Negative Bacteria

Gram-negative bacteria maintain zinc homeostasis through coordinated uptake, efflux, storage, and Zur-mediated regulatory systems, enabling adaptation to host nutritional immunity and highlighting zinc homeostasis as a potential antimicrobial target (Table 2).

Gram-negative bacteria employ a multifaceted regulatory network to maintain intracellular zinc homeostasis. They employ distinct zinc uptake systems according to zinc availability. In most Gram-negative bacteria, the low-affinity ZIP-family transporter ZupT mediates broad-spectrum metal uptake when zinc is relatively available, whereas the high-affinity ZnuABC transporter is induced under zinc-limited conditions to support zinc acquisition [43,81]. ZnuABC is a high-affinity zinc uptake system conserved in most Gram-negative bacteria. However, canonical ZnuABC systems have not been identified in several atypical diderm pathogens, including *Cupriavidus metallidurans*, *Neisseria* species, *Chlamydia* species, and *Borrelia* species, in which metal acquisition instead involves alternative or incompletely characterized transport pathways [83,84,85]. For example, pathogenic *Neisseria* species rely on TonB-dependent outer-membrane receptors for zinc acquisition [86]. ZnuABC consists of ZnuA, a periplasmic Zn (II)-binding protein; ZnuB, a cytoplasmic membrane permease that mediates Zn (II) translocation; and ZnuC, a cytoplasmic ATPase that energizes transport through ATP hydrolysis [87,88]. In species that encode ZnuD, this TonB-dependent outer-membrane receptor contributes to zinc acquisition across the outer membrane under zinc-limited conditions, thereby supplying periplasmic Zn (II) for subsequent ZnuABC-mediated import [86]. Together, these components mediate high-affinity zinc acquisition and enable Gram-negative bacteria to adapt to host-imposed zinc limitation. At the transcriptional level, zinc uptake regulator Zur functions as a Zn (II)-responsive repressor that coordinates bacterial zinc acquisition [82,89]. When intracellular zinc is sufficient, Zur binds Zur-box motifs in the promoter regions of target genes, such as *znuABC*, and represses their transcription to limit excessive zinc influx. Under zinc-limited conditions, reduced zinc availability weakens Zur–DNA binding, thereby relieving repression and inducing the expression of zinc acquisition systems. This derepression process induces a coordinated zinc-starvation response, including the expression of high-affinity zinc uptake systems such as ZnuABC, species-specific metallophore-mediated zinc acquisition pathways such as the pseudopaline-associated ZrmABCD system in *P. aeruginosa*, and zinc-sparing proteins that replace selected zinc-dependent proteins with zinc-independent paralogues [45,90,91].

Metallophores are distributed across a range of bacterial taxa, including both Gram-negative and Gram-positive pathogens. Within Gram-negative taxa, metallophore-mediated zinc acquisition represents a lineage- and niche-specific strategy by which some Gram-negative bacteria adapt to host-imposed zinc restriction during nutritional immunity. Zincophores are bacterially secreted metallophores that chelate zinc under zinc-limited conditions and facilitate uptake of zinc-ligand complexes. During infection, this strategy is particularly relevant because host metal-sequestering proteins, such as calprotectin, markedly reduce free zinc availability. Bioinformatic analyses have suggested that opine-like zincophore biosynthetic gene clusters are distributed across selected bacterial taxa, including some Proteobacteria [92]. In organisms that encode these systems, zincophores can enable pathogens to mobilize extracellular or host-bound zinc pools that would otherwise remain poorly accessible [93]. One well-characterized Gram-negative zinc metallophore is pseudopaline, produced by *P*. *aeruginosa*. Pseudopaline is an opine- or nicotianamine-related metallophore encoded by the *zrmABCD* locus, also referred to as *cntOLMI* [91,94]. Yersiniabactin, originally characterized as an iron-scavenging siderophore, can function as a zincophore in selected Enterobacteriaceae by binding zinc, counteracting calprotectin-mediated zinc sequestration, and promoting *E. coli* colonization of the inflamed gut as well as *Yersinia pestis* infection of mammalian and insect hosts [95,96]. Recent evidence has expanded the role of pyochelin beyond iron acquisition. In *P. aeruginosa*, pyochelin-mediated cobalt import through Zur-regulated transport systems can reduce zinc demand and support growth under severe zinc limitation [90].

To counteract zinc overload, Gram-negative bacteria deploy three primary efflux systems: P-type ATPases, resistance-nodulation-cell division (RND) transporters, and cation diffusion facilitator (CDF) family proteins, which upregulate zinc-export machinery during high zinc stress [50,97]. P-type ATPases such as ZntA/CadA export excess cytoplasmic zinc in an ATP-dependent manner, whereas CDF proteins such as ZitB/YiiP are usually located in the inner membrane and export excess cytoplasmic zinc or other divalent metals through metal/proton antiport [98]. RND-type systems such as CzcCBA form tripartite envelope-spanning complexes that export Zn (II), Cd (II), and Co (II) across the Gram-negative cell envelope [99]. These systems not only maintain intracellular zinc equilibrium but also contribute to multidrug resistance through RND-mediated xenobiotic extrusion, with expelled zinc ions immobilized extracellularly via protein binding or precipitation to prevent reuptake [100]. In *P*. *aeruginosa*, zinc homeostasis intersects with quorum sensing through the zinc-responsive regulator CzcR. CzcR activates CzcCBA, an RND-type zinc-efflux system, and also regulates *lasI*, thereby promoting Las/Rhl autoinducer production and quorum-sensing-controlled phenotypes such as elastase and rhamnolipid production and biofilm formation [101]. Consistent with the susceptibility of these signaling networks to zinc-related perturbation, exogenous zinc oxide nanospikes have also been shown to suppress Las- and Pqs-associated quorum sensing and reduce downstream virulence-factor production and biofilm formation [102].

Concurrently, zinc storage proteins like MTs buffer intracellular zinc by sequestering excess ions to mitigate toxicity, while retaining capacity for controlled release during metabolic demands [103]. In Gram-negative bacteria other than cyanobacteria, MTs have been most clearly characterized in the genus *Pseudomonas*, where MT homologues are conserved in many species, including *P. aeruginosa*, *Pseudomonas fluorescens*, and *Pseudomonas putida* [104]. In *P. aeruginosa*, PmtA contributes to oxidative stress adaptation and virulence-related traits, including pyocyanin production, biofilm formation, antibiotic susceptibility, and resistance to macrophage phagocytosis and innate immune pressure [105,106].

### 3.3. Pathogen-Specific Zinc Homeostasis in Clinically Important Gram-Negative Bacteria

Although ZupT, ZnuABC, zinc efflux systems, and Zur-mediated transcriptional control represent conserved modules of bacterial zinc homeostasis, clinically important Gram-negative pathogens have evolved species-specific strategies to adapt to distinct host niches and immune pressures (Table 3).

In *P. aeruginosa*, zinc homeostasis is maintained by multiple uptake and efflux systems. Under zinc-limited conditions, *P. aeruginosa* can employ the ZnuABC transporter and the pseudopaline-associated ZrmABCD system to compete with the host for zinc [108]. Conversely, under zinc overload, *P. aeruginosa* mainly relies on the CadA P-type ATPase and the CzcCBA RND efflux pump. CadA is rapidly induced in response to excess zinc and facilitates timely activation of the CzcRS regulatory system [51]. Notably, activation of CzcRS not only induces the CzcCBA efflux pump but also represses the *oprD* porin gene, thereby reducing carbapenem uptake and linking zinc stress adaptation to carbapenem resistance. In *K. pneumoniae*, zinc acquisition depends on two high-affinity ABC permeases, ZnuABC and ZniABC. ZnuABC is the canonical and broadly conserved Gram-negative zinc uptake system, whereas ZniABC represents a Klebsiella-associated Zn (II) importer that is strongly induced under zinc-limited conditions. These two permeases appear functionally redundant, as deletion of either *znuA* or *zniA* alone has limited effects on zinc accumulation, whereas simultaneous loss of both systems markedly impairs zinc uptake [39]. Zinc efflux constitutes a critical determinant of *K. pneumoniae* fitness. The P-type ATPase ZntA is strongly induced under zinc stress and functions as a major zinc detoxification pathway. Loss of *zntA* leads to impaired growth under zinc overload, increased intracellular zinc accumulation, dysregulation of manganese and iron homeostasis, and altered biofilm formation [110].

In addition to ZnuABC, *A*. *baumannii* induces the Zur-regulated peptidase ZrlA to preserve cell wall and envelope integrity during immune-mediated zinc sequestration. Loss of *zrlA* impairs bacterial growth under zinc-limited conditions, increases cell envelope permeability, alters peptidoglycan composition, and enhances antibiotic efficacy in vitro and in a murine pneumonia model [117]. In *S. enterica* serovar Typhimurium, the high-affinity ZnuABC transporter and low-affinity ZupT transporter cooperatively support zinc acquisition under metal-limited conditions, and their simultaneous loss causes severe growth defects, oxidative stress sensitivity, and reduced colonization capacity [121]. Zinc efflux systems, including the P-type ATPase ZntA and the CDF family transporter ZitB, protect bacteria from zinc overload and contribute to metal stress resistance during infection. Deletion of *zntA*, *zitB*, or both impairs bacterial survival and virulence in mouse infection models, highlighting the importance of zinc detoxification during host–pathogen interactions [120].

Pathogenic *Neisseria* species, particularly *Neisseria meningitidis* and *Neisseria gonorrhoeae*, acquire zinc from host zinc-binding proteins through TonB-dependent outer membrane receptors, representing a strategy distinct from the classical ZnuABC-centered model. In *N. meningitidis*, zinc limitation induces the outer membrane receptor CbpA, which binds calprotectin and enables the bacterium to use calprotectin as a zinc source in a TonB-dependent manner [127]. Similarly, *N. gonorrhoeae* produces the TonB-dependent transporter TdfH, which is regulated by zinc and Zur, binds human calprotectin, promotes zinc accumulation, and enhances survival during exposure to neutrophil extracellular traps (NETs) [124]. This “zinc piracy” strategy allows pathogenic *Neisseria* to subvert calprotectin-mediated nutritional immunity. Therefore, pathogen-specific zinc homeostasis pathways may provide more precise antimicrobial targets than broadly disrupting zinc metabolism.

## 4. The Role and Mechanism of Zinc in Host Infection by Gram-Negative Bacteria

### 4.1. The Role of Zinc in the Pathogenicity of Gram-Negative Bacteria

**Figure 3:**
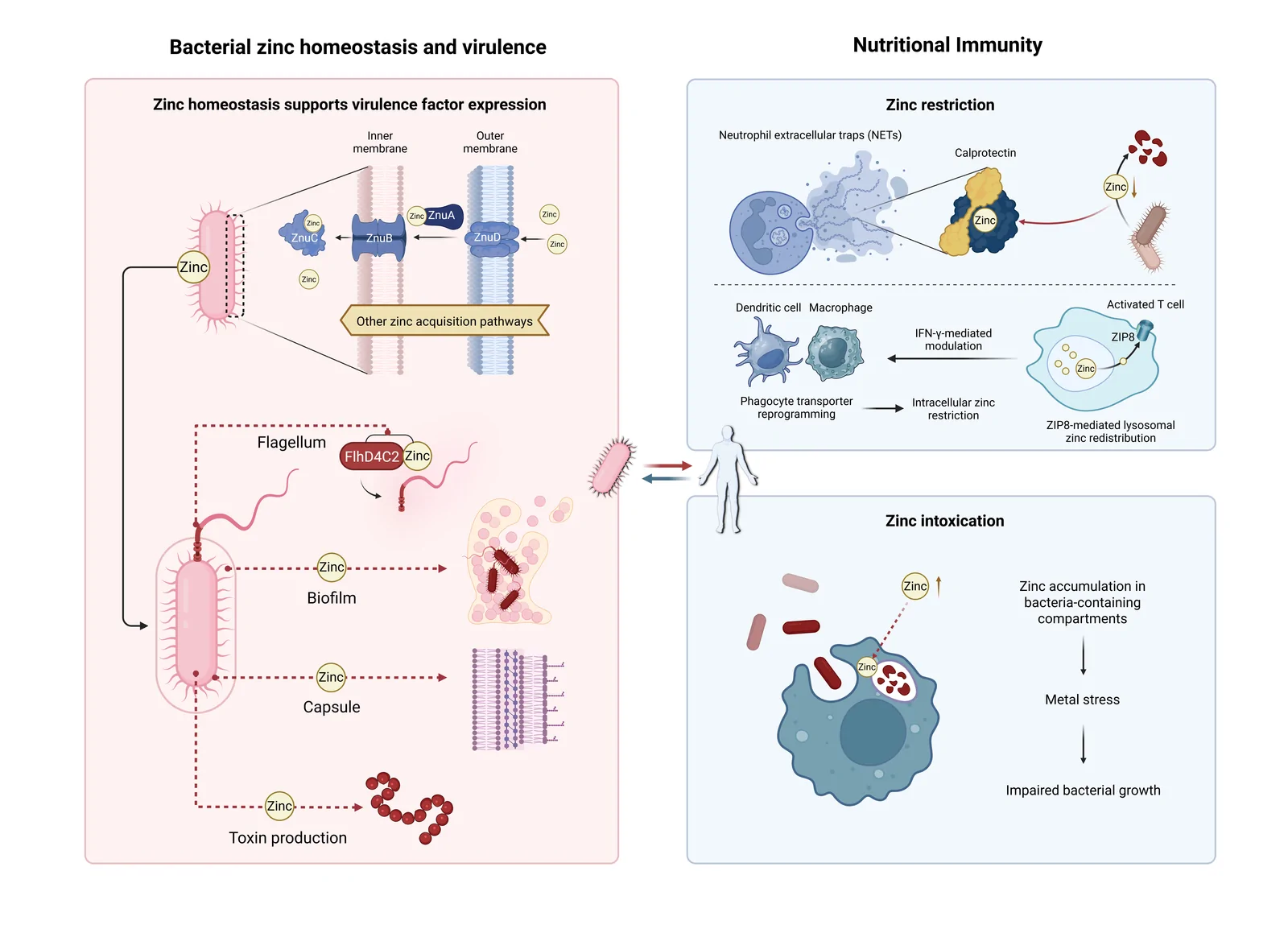
The role of zinc in the pathogenicity of Gram-negative bacteria and host immune response. In Gram-negative bacteria, zinc acquisition through the ZnuABC transporter, the TonB-dependent outer-membrane receptor ZnuD, and other uptake pathways supports cellular fitness and virulence-associated traits, including flagellar function, biofilm formation, capsule production, and toxin expression. In the host, zinc contributes to nutritional immunity through coordinated zinc restriction and zinc intoxication. The restriction arm operates at both extracellular and intracellular levels. Extracellularly, neutrophils release neutrophil extracellular traps (NETs) containing metal-sequestering proteins such as calprotectin, which binds zinc ions and reduces its availability to invading bacteria. Intracellularly, macrophages and dendritic cells reprogram zinc import and export pathways to decrease zinc availability within pathogen-accessible cellular compartments. In activated T cells, upregulated ZIP8 transports zinc from lysosomes into the cytoplasm, thereby depleting lysosomal zinc pools. The resulting increase in cytoplasmic zinc also promotes IFN-γ production, which modulates macrophage and dendritic-cell antimicrobial responses. Conversely, zinc intoxication promotes zinc accumulation within bacteria-containing compartments, thereby inducing metal stress and inhibiting bacterial growth.

Zinc plays multifaceted roles in bacterial virulence, serving as an essential trace element for bacterial survival and modulating the expression of other virulence factors directly or indirectly, which in turn impacts bacterial pathogenicity (Figure 3).

Zinc availability directly influences the pathogenic fitness of Gram-negative bacteria during infection. Its transport system, particularly the ZnuABC system, contributes to bacterial colonization and virulence-associated phenotypes by maintaining zinc homeostasis during infection [128]. Its deletion severely compromises virulence in multiple Gram-negative pathogens. For instance, in a *K. pneumoniae* pulmonary infection model, double knockout of ZnuABC and ZniABC systems resulted in markedly reduced bacterial virulence [39]. In *P. aeruginosa*, deletion of *znuA* leads to impaired alginate production, reduced extracellular zinc-dependent protease activity, and attenuated dissemination during infection [129]. Calprotectin-mediated zinc and manganese sequestration restricts *A*. *baumannii* growth during lung infection while the ZnuABC zinc acquisition system enables resistance to this metal-limiting pressure. Deletion of *znuB* reduces bacterial fitness in pulmonary infection models and limits dissemination from the primary infection site [113]. Similarly, *S*. *enterica* serovar Typhimurium strains with a *znuA* gene knockout maintained normal growth in zinc-replete conditions but showed impaired proliferation and significantly reduced virulence under zinc limitation [38]. As a *P. aeruginosa*-specific example, pseudopaline promotes bacterial growth and zinc uptake under zinc-poor or chelating conditions. Deletion of *cntL*, a gene involved in pseudopaline biosynthesis, reduced intracellular zinc accumulation by approximately 60% in EDTA-treated medium. As a result, perturbation of zinc homeostasis attenuated *P. aeruginosa* pathogenicity in mouse models of acute lung and systemic infection [91,130].

In addition to its direct contribution to bacterial fitness, zinc homeostasis can affect bacterial pathogenicity by modulating the expression or activity of established virulence-associated factors. The flagellum is a key virulence factor for *Proteus mirabilis* to enter and colonize the host urinary tract [131]. FlhD4C2, the primary regulator of flagella, contains a putative zinc-binding site [132]. In *znuC* mutants, the expression of flagella was significantly reduced, indicating a crucial role of zinc in flagella biosynthesis [42]. Meanwhile, zinc is essential for the development and maintenance of biofilms, which protect bacteria from immune attack and antibiotics on host surfaces. In zinc-limited conditions, the ZnuABC system in enterotoxigenic *E. coli* (ETEC) F4ac enables its biofilm stability and promotes adhesion to host cells. In *P. aeruginosa*, this species-specific Cnt/pseudopaline-mediated zinc acquisition supports multiple infection-associated phenotypes, as disruption of the Cnt pathway impaired mature biofilm formation, increased biofilm susceptibility to tobramycin, reduced survival-related phenotypes during macrophage interaction, and attenuated virulence in a murine lung infection model [133]. The bacterial capsule, a polysaccharide layer on the cell surface, shields bacteria from host immune defenses while augmenting bacterial virulence [134]. In *K. pneumoniae*, zinc deprivation downregulated the regulatory protein ChaB, with *chaB* gene knockout mutants exhibiting less capsular production and attenuated pathogenicity [135]. These findings further establish zinc’s important role in capsule formation and bacterial virulence. The enterotoxin, a virulence factor secreted by Gram-negative bacteria, can induce intestinal pathology in hosts [136]. Under marginal zinc deficiency, relative expressions of virulence genes associated with enterotoxins, motility, cell adhesion, and biofilm formation were upregulated in cecal contents of mice infected with ETEC [137]. These genetic responses exacerbated diarrhea, intestinal barrier dysfunction, and inflammation caused by ETEC K88 infection, demonstrating that zinc deficiency may potentiate ETEC virulence through enhanced pathogenic gene expression. A similar phenomenon, in which zinc limitation enhances bacterial pathogenic potential, has also been observed in uropathogenic *E*. *coli*. Zinc limitation relieves Zur-dependent repression of the hlyII operon, leading to increased α-hemolysin expression and enhanced cytotoxic responses in host cell models [138].

### 4.2. The Role of Zinc in the Host Immune Response Against Gram-Negative Bacterial Infection

Zinc serves as a pivotal factor in the host immune system to combat Gram-negative bacterial infections (Figure 3). The host restricts bacterial access to zinc while deploying zinc-dependent immune mechanisms to support antimicrobial defense. To curb bacterial growth and colonization, the host manipulates zinc availability through nutritional immunity, which includes both zinc intoxication and zinc restriction.

Zinc cytotoxicity or intoxication is characterized by metal stress-induced damage, arising when excessive zinc accumulates within bacteria-containing compartments. The host immune cells can directly kill or inhibit bacteria by increasing intracellular zinc ion concentrations [56]. For example, macrophages elevate intracellular zinc levels to toxic thresholds after phagocytosing bacteria. Excess zinc ions can then damage the normal structure and function of bacteria cells via oxidative stress or by disrupting metabolic pathways [139,140]. When *E. coli* primarily resides within membrane-bound vesicular compartments in macrophages, macrophages upregulate LPS-inducible SLC30A1 from cytoplasm to transport zinc into lumen of vesicles, increasing zinc concentration and inducing zinc toxicity against intracellular *E. coli* [141]. Uropathogenic *E. coli* subverts this innate immune-mediated zinc toxicity by evading zinc-enriched antibacterial compartments and relying on zinc detoxification systems, particularly ZntA, to promote intracellular survival and systemic dissemination [142]. More recently, cystic fibrosis transmembrane conductance regulator (CFTR) has also been shown to be required for macrophage-mediated zinc intoxication by promoting zinc accumulation and zinc vesicle formation. SLC30A1 expression or zinc supplementation partially restoring antibacterial activity against both *E. coli* and *P*. *aeruginosa* in CFTR-defective macrophage models [143]. Zinc cytotoxicity also intersects with nitrosative stress to amplify antibacterial pressure. In *S*. *enterica* serovar Typhimurium, macrophage-derived nitric oxide induces endogenous zinc intoxication by mobilizing zinc from bacterial metalloproteins, increasing the labile zinc pool and exacerbating metal stress [54].

An important component of nutritional immunity is metal restriction, whereby the host limits bacterial access to essential micronutrients, including iron, zinc and manganese, at the host–pathogen interface [8]. This process involves metal sequestration, redistribution, and regulated metal transport to reduce the bioavailability of these nutrients to invading bacteria. Extracellular zinc limitation is mainly achieved by secreted metal-binding proteins, such as S100 family proteins [144,145]. Neutrophils contribute to this process by deploying NETs to release metal-sequestering proteins such as calprotectin and lactoferrin, thereby creating metal-limited conditions, such as a zinc-deficient microenvironment, that suppress bacterial growth and virulence [146]. Calprotectin, a neutrophil- and epithelial cell-derived S100A8/S100A9 heterodimer, sequesters multiple divalent transition metals at sites of inflammation, including Zn (II), Mn (II), and Fe (II) [147]. In *A*. *baumannii*, calprotectin-mediated Zn/Mn limitation contributes to host defense during pneumonia, and the bacterial ZnuABC system is required to overcome host-imposed zinc starvation [148]. In *S*. *enterica* serovar Typhimurium, neutrophil-derived calprotectin restricts zinc availability in the inflamed gut, forcing the bacteria to rely on high-affinity zinc acquisition systems to maintain growth under metal-limited conditions [149]. Other S100 proteins like calgranulin-C (S100A12) can inhibit *Helicobacter pylori* and *C*. *jejuni* growth in a zinc-dependent manner [150,151]. Cytokines such as IL-22 and IL-17 further amplify nutritional immunity by inducing epithelial antimicrobial proteins, including calprotectin and lipocalin-2 [152,153]. These responses restrict microbial access to zinc, manganese and iron, but *S*. *enterica* serovar Typhimurium can benefit because they suppress susceptible commensal Enterobacteriaceae more strongly than *S*. *enterica*. Concurrently, both innate immune cells and adaptive immune cells are involved in intracellular zinc restriction. Macrophages, dendritic cells, and other phagocytes exert dual regulation of zinc import and export pathways through transporter reprogramming to minimize cytoplasmic zinc availability [154,155]. Activated T cells secrete cytokines like IFN-γ to modulate macrophages and dendritic cells [156]. During T cell activation, ZIP8 (SLC39A8) which primarily localized to the lysosome, is upregulated and transports zinc from lysosomes to the cytoplasm, depleting lysosomal zinc and restricting bacterial zinc acquisition [157]. Meanwhile, the increased cytoplasmic zinc inhibits calcineurin activity, leading to increased phosphorylation of the transcription factor cAMP-response element-binding protein (CREB). This further promotes IFN-γ expression, thereby enhancing the host’s immune defense [157,158].

## 5. Emerging Metal-Targeted Strategies for Antibacterial Therapy

The growing threat of multidrug-resistant bacteria has spurred investigations into unconventional therapeutic targets, particularly metal homeostasis. Given the intense competition for zinc at the host–pathogen interface, manipulating bacterial zinc homeostasis offers a potent strategy to compromise virulence and survival. Promising approaches include targeting zinc transport systems, inhibiting zinc-dependent enzymes, modulating local metal availability, and fabricating advanced zinc-based antimicrobial materials.

A direct strategy is to interfere with bacterial zinc uptake and efflux systems, thereby amplifying host-imposed zinc sequestration and metal intoxication. In localized *P. aeruginosa* infection models, strains defective in the high-affinity ZnuABC and pseudopaline-ZrmABCD zinc uptake systems showed reduced colonization at later stages of infection. Meanwhile, the early induction of the host ZnT-family transporters SLC30A1A and SLC30A4 suggested that zinc redistribution may contribute to antimicrobial intoxication [159]. Genetic disruption of zinc-acquisition systems can attenuate infection, but direct pharmacological validation of these targets remains limited. Nevertheless, early studies suggest that bacterial zinc uptake can be pharmacologically impaired by directly targeting individual transporter components. An early proof-of-concept study identified the diaryl-pyrrole hydroxamic acids RDS50 and RDS51 as ZnuA-targeting compounds that inhibited *S. enterica* serovar Typhimurium growth and reduced bacterial invasion of epithelial cells. The ZnuA–Zn(II)–RDS51 structure suggested that RDS51 stabilizes Zn(II) within the binding site, thereby hindering zinc release from ZnuA and transfer to the membrane transporter [87]. Structural characterization of the TonB-dependent outer-membrane zinc receptor ZnuD has supported its evaluation as a vaccine target, and in *A. baumannii*, a hybrid antigen displaying four surface-exposed ZnuD loops conferred complete protection in a murine sepsis model, although whether this effect involved direct inhibition of zinc uptake remains unclear [160].

Beyond the major zinc transport systems, accessory or regulatory proteins and zinc-dependent enzymes provide additional therapeutic targets. Recent work in *N. gonorrhoeae* identified Zcp, a periplasmic zinc capture protein from the DUF4198 family, as an important contributor to envelope integrity, maximal growth and infectivity toward epithelial cells and neutrophils under zinc limitation [126]. Importantly, Zcp is widely distributed among Gram-negative bacteria and deletion of *zcp* increased sensitivity to envelope-targeting antimicrobials, suggesting that zinc-homeostasis-associated proteins may be exploited to weaken bacterial adaptation during nutritional immunity. Another promising direction is inhibition of bacterial zinc-dependent enzymes. Several Gram-negative bacterial zinc metalloenzymes, including LpxC, thermolysin-like enzymes, and pseudolysin, represent potential antibacterial or antivirulence targets, but their therapeutic development requires improved selectivity, Gram-negative envelope permeability, and strategies to limit resistance development [161].

Metal availability can also be manipulated pharmacologically. Metal ionophore-mediated delivery has been investigated as an antibiotic-adjuvant strategy. Zinc ionophores promote intracellular zinc accumulation and can be used to induce metal dyshomeostasis. PBT2, a second-generation 8-hydroxyquinoline derivative originally developed for neurodegenerative diseases, functions as a zinc ionophore and has been shown to restore the susceptibility of polymyxin-resistant Gram-negative pathogens, including *K. pneumoniae*, *E. coli*, *A. baumannii*, and *P. aeruginosa*, to polymyxin-class antibiotics [55]. In multidrug-resistant *A. baumannii*, PBT2 combined with zinc resensitized bacteria to tetracycline-class antibiotics, including tetracycline, doxycycline, and tigecycline, and showed efficacy in a murine pulmonary infection model [162]. Another zinc ionophore is zinc pyrithione, which enhanced intracellular zinc delivery, inhibited the aminoglycoside resistance enzyme AAC(6′)-Ib, and restored amikacin activity against resistant *A. baumannii* and *E. coli* in vitro [163]. Although these findings support ionophore-mediated zinc delivery as a promising antibiotic-adjuvant strategy, its clinical translation remains limited by insufficient in vivo validation and unresolved concerns regarding metal selectivity, pharmacokinetics, toxicity, and potential disruption of host metal homeostasis.

Finally, zinc-based antimicrobial materials provide a complementary strategy that is particularly suitable for local or device-associated applications. Zinc oxide nanoparticles and zinc sulfide nanoparticles have been shown to reduce planktonic growth and biofilm formation in clinically relevant bacteria, including *Klebsiella oxytoca* and *P. aeruginosa* [164]. These findings support zinc-based nanoparticles as potential local antibiofilm agents, particularly for wound-infection management and antimicrobial coatings, but safe dosing, biocompatibility, delivery, and metal-tolerance risks require further evaluation.

Collectively, these studies demonstrate that bacterial zinc homeostasis can be disrupted through several experimentally investigated approaches. However, most strategies remain at the proof-of-concept or preclinical stage. Further development will therefore require selective lead optimization, validation in clinically relevant infection models, and careful assessment of bacterial specificity, host toxicity, pharmacokinetics, and resistance potential.

## 6. Conclusions

In summary, we comprehensively elucidate the multifaceted roles of zinc in host–microbe interactions, particularly in the context of multidrug-resistant Gram-negative bacterial infections. The interaction between the host and bacteria is a complex battle involving the intricate regulation of zinc ions. The host employs nutritional immunity, including both zinc restriction and zinc cytotoxicity, while bacteria counteract these pressures through adaptive zinc acquisition, storage, and detoxification mechanisms. Despite the detailed mechanisms described, the precise interplay between host zinc-dependent defenses and bacterial adaptive responses remains incompletely understood. By highlighting the critical roles of zinc in both host defense and bacterial virulence, it underscores the potential for targeting zinc homeostasis as a novel therapeutic strategy, which is promising but still in its infancy. Therefore, further research is needed to translate these findings into effective clinical treatment methods.

## Figures and Tables

**Table 1:** The Fundamental Components of Zinc Homeostasis Regulation in the Host.

Structure		Function	Reference
Metal regulatory transcription factor 1(MTF-1)		Zinc ion sensor involved in gene expression	[79]
Zinc transporters	SLC39s family (ZIP 1–14)	Increase cytoplasmic zinc ion concentration	[68]
	SLC30s family (ZnT 1–10)	Reduce cytoplasmic zinc ion concentration	
Metallothionein (MT)		Zinc donor and acceptor for zinc buffering	[72]
Zincosome		Cellular vesicles for zinc storage	[73,80]

**Table 2:** The Fundamental Components of Zinc Homeostasis Regulation in Gram-Negative Bacteria.

Structure		Function	Reference
Zinc uptake systems	ZupT transporter	Low-affinity zinc importer under zinc sufficiency with broad substrate specificity	[43,81]
	ZnuABC complex transporter	High-affinity tripartite zinc importer under zinc deficiency	
Zinc efflux systems	P-type ATPase	Facilitate Zn (II) efflux under high zinc conditions to maintain cytoplasmic zinc balance	[50]
	RND transporter		
	CDF family protein		
Metallothionein (MT)		Sequester excess Zn (II) for intracellular zinc storage and buffering	[12]
Zinc uptake regulator (Zur)		Zinc-sensing transcriptional regulator of zinc uptake gene expression	[82]

**Table 3:** Zinc Homeostasis Strategies in Representative Gram-negative Pathogens.

Pathogen	Zinc Acquisition Systems	Pathogen-Specific Acquisition Features	Reference	Zinc Efflux Systems	Pathogen-Specific Efflux Features	Reference
*P . aeruginosa*	ZnuABC	Zur-regulated high-affinity ABC transporter for Zn (II) uptake.	[107]	CadA	CadR-regulated P-type ATPase for rapid cytoplasmic Zn (II) efflux.	[51]
	ZnuD-like transporter	Putative TonB-dependent outer-membrane transporter for Zn (II) entry across the outer membrane into the periplasm.	[107]	CzcCBA	CzcRS-regulated RND efflux pump for high-capacity Zn (II)/Cd (II)/Co (II) export.	[108]
	ZrmABCD	Pseudopaline-mediated zincophore system for Zn (II) acquisition under metal limitation.	[91]	CzcD	CDF-family transporter contributing to zinc tolerance and metal homeostasis.	[109]
*K . pneumoniae*	ZnuABC	Zur-regulated high-affinity ABC transporter for Zn (II) uptake.	[39]	ZntA	Primary P1B-type ATPase for cytoplasmic Zn (II) and Cd (II) efflux.	[110]
	ZniABC	Klebsiella-conserved second ABC permease redundant with ZnuABC for Zn (II) acquisition.	[39]	ZitB	Conserved CDF-family putative Zn (II) exporter with no definitive role.	[110]
	ZupT	Conserved ZIP-family putative low-affinity Zn (II) importer.	[39]	FieF	Conserved CDF-family Fe (II)/Zn (II) transporter with undefined role.	[110]
	ZinT	Putative periplasmic metallochaperone associated with Zn (II) recruitment during zinc limitation.	[111]	ZntB	Conserved CorA-family zinc transporter with ambiguous import/export directionality.	[112]
*A . baumannii*	ZnuABC	Zur-regulated high-affinity ABC transporter for Zn (II) uptake.	[113]	CzcICBAD/CzcCBA	RND-type heavy-metal efflux system providing primary Zn (II) resistance.	[114]
	ZnuD1	Zur-regulated TonB-dependent outer-membrane receptor implicated in Zn (II) entry.	[115]	CzcA	Inner-membrane RND component required for high-capacity Zn (II) efflux.	[116]
	ZnuD2	Plasmid-encoded ZnuD-like outer-membrane receptor induced under zinc limitation.	[115]	CzcB/CzcC	Periplasmic adaptor and outer-membrane channel of the CzcCBA efflux pump.	[116]
	ZrlA	Zur-regulated Zn (II)-binding peptidase maintaining cell envelope integrity during zinc limitation.	[117]	CzcD	CDF-family transporter contributing to cytoplasmic Zn (II) export and zinc tolerance.	[116]
	ZigA	COG0523-family Zn (II) metallochaperone required during zinc starvation.	[118]	CzcI	Putative periplasmic metal-binding accessory protein linked to CzcCBA induction.	[116]
*S . enterica*	ZnuABC	Zur-regulated high-affinity ABC transporter for Zn (II) uptake.	[119]	ZntA	ZntR-regulated P1B-type ATPase for Zn (II)/Cd (II) efflux.	[120]
	ZupT	Conserved ZIP-family low-affinity Zn (II) importer.	[121]	ZitB	CDF-family Zn (II) exporter contributing to zinc-stress resistance.	[120]
	ZinT	Zur-regulated periplasmic metallochaperone assisting ZnuABC-mediated Zn (II) recruitment.	[111]	FieF	CDF-family Fe (II)/Zn (II) transporter with a secondary role in zinc resistance.	[120]
	PitA	Phosphate-linked low-affinity route potentially associated with Zn (II) uptake.	[119]	ZntB	CorA-family transporter with demonstrated Zn (II) transport activity.	[122]
*Neisseria* spp.	ZnuABC	Zur-regulated high-affinity ABC transporter for Zn (II) uptake.	[123]	-		
	ZnuD	Zur-regulated outer-membrane receptor for Zn (II) uptake	[86]			
	TdfH/CbpA	Calprotectin-binding TonB-dependent receptor mediating host zinc piracy	[124,125]			
	Zcp	Periplasmic zinc-capture protein enhancing growth under zinc limitation	[126]			

## Data Availability

No new data were created or analyzed in this study. Data sharing is not applicable to this article.

## Abbreviations

ATPase	Adenosine triphosphatase
CDF	Cation diffusion facilitator
CFTR	Cystic fibrosis transmembrane conductance regulator
CREB	cAMP-response element-binding protein
ETEC	Enterotoxigenic *Escherichia coli*
HDAC	Histone deacetylase
LCK	Lymphocyte-specific protein tyrosine kinase
LPS	Lipopolysaccharide
MIC	Minimum inhibitory concentration
MRE	Metal response element
MT	Metallothionein
MTF-1	Metal-regulatory transcription factor 1
NADPH	Nicotinamide adenine dinucleotide phosphate hydrogen
NET	Neutrophil extracellular trap
RND	Resistance-nodulation-cell division
ROS	Reactive oxygen species
SLC30/39	Solute carrier family 30/39
SOD	Superoxide dismutase
TCR	T-cell receptor
TLR4	Toll-like receptor 4
WHO	World Health Organization
ZFP	Zinc finger protein
ZIP	Zrt-Irt-like protein
ZnT	Zinc transporter
ZnuABC	Zinc uptake ATP-binding cassette transporter
ZupT	Zinc uptake transporter
Zur	Zinc uptake regulator
